# Preliminary Results of Universiti Sultan Zainal Abidin Community Bone Health Screening in Manir, Kuala Terengganu using Bone Densitometry Calcaneal Quantitative Ultrasound (QUS) in Conjunction with Clinical Risk Factors

**DOI:** 10.5704/MOJ.2307.014

**Published:** 2023-07

**Authors:** Kamudin NAF, Ibrahim MS, Mohamed-Yusoff H

**Affiliations:** 1 Department of Orthopaedics, Universiti Sultan Zainal Abidin (UniSZA), Kuala Terengganu, Malaysia; 2 Department of Clinical Education, Universiti Sultan Zainal Abidin (UniSZA), Kuala Terengganu, Malaysia; 3 Department of Family Medicine, Universiti Sultan Zainal Abidin (UniSZA), Kuala Terengganu, Malaysia

Dear Editor,

I am writing to bring attention to the issue of fragility fractures in the growing aged population of Malaysia. According to the International Osteoporosis Foundation, up to 70% of people are at risk and underdiagnosed with osteoporosis^1^. This is a significant public health concern that needs to be addressed. Central Dual-energy X-ray Absorptiometry (DXA) is the gold standard for diagnosing osteoporosis, but unfortunately, not all populations have access to this facility^2^.

To address this issue, we used bone densitometry calcaneal quantitative ultrasound (QUS) with a fracture risk assessment tool (Frax-tool) to screen for bone health. This will allow for identification of population with low and high probability of osteoporotic fractures^3^. A health screening was conducted in Manir, Kuala Terengganu during a two-day local public event. The screening consisted of three stations: registration for profile and lifestyle data collection, screening QUS with three total readings and Frax-tool, and the final station for counselling by an expert. A total of 120 patients completed the screening. Our results showed that poor bone health was detected among 39 (32.5%) screened by both QUS and Frax-tool, 25 (20.8%) by QUS only, 15 (12.5%) by Frax-tool only, and the remaining 41 (34.2%) were normal. This means that one-third of the total screened population was at risk of osteoporosis, fragility fractures, and all other associated complications, signifying a huge volume of benefits from early detection.

We also found that QUS is highly consistent in detecting hip fragility fractures, especially for females over 50 years old, but not for other locations (spine, wrist, or humerus)/ major osteoporosis. Therefore, we recommend a combined QUS and Frax-tool for community-based bone health screening. Clinicians may choose to draw a high-risk conclusion whenever the readings disagree. Moreover, our study showed an excellent agreement in the intraclass correlation coefficient for the three QUS readings, which was 0.962 (95% CI: 0.949, 0.973, p<0.001). This indicates that the highly reliable QUS reading need not be repeated in most cases.

In conclusion, we believe that community-based bone health screening is essential for the early detection and prevention of fragility fractures, especially in populations where access to central DXA facilities is limited^4^. We recommend a combined QUS and Frax-tool for osteoporosis screening, and future studies may include a DEXA scan to determine the increase in sensitivity and specificity of screening using the dual-modality approach. We hope that our findings will contribute to a better understanding and management of osteoporosis in Malaysia, and we urge policymakers to consider implementing community-based bone health screening programs to improve the quality of life of our aging population.

## References

[1] Mithal A, Ebeling P, Kyer CS (2013). The Asia-Pacific Regional Audit - Epidemiology, Costs, and Burden of Osteoporosis in 2013: A report of International Osteoporosis Foundation 201311-1500.. https://www.osteoporosis.foundation/sites/iofbonehealth/files/2019-06/2013_Asia_Pacific_Audit_English.pdf.

[2] de Villiers TJ, Goldstein SR (2022). Bone health 2022: an update.. Climacteric..

[3] (. a) Adami G, Arioli G, Bianchi (b) G, Brandi ML, Caffarelli C, Cianferotti L (2020). Radiofrequency echographic multi spectrometry for the prediction of incident fragility fractures: A 5-year follow-up study.. Bone..

[4] Hans D, Métrailler A, Gonzalez Rodriguez E, Lamy O, Shevroja E (2022). Quantitative Ultrasound (QUS) in the Management of Osteoporosis and Assessment of Fracture Risk: An Update.. Adv Exp Med Biol..

